# Automatic Quantification of Atherosclerosis in Contrast-Enhanced MicroCT Scans of Mouse Aortas Ex Vivo

**DOI:** 10.1155/2021/4998786

**Published:** 2021-09-20

**Authors:** Vincent A. Stadelmann, Gabrielle Boyd, Martin Guillot, Jean-Guy Bienvenu, Charles Glaus, Aurore Varela

**Affiliations:** ^1^Department of Research and Development, Schulthess Klinik, Zurich, Switzerland; ^2^Charles River Laboratories Montreal ULC, Senneville, QC, Canada; ^3^Amgen Research, Amgen Inc, Thousand Oaks, CA, USA

## Abstract

**Objective:**

While microCT evaluation of atherosclerotic lesions in mice has been formally validated, existing image processing methods remain undisclosed. We aimed to develop and validate a reproducible image processing workflow based on phosphotungstic acid-enhanced microCT scans for the volumetric quantification of atherosclerotic lesions in entire mouse aortas. *Approach and Results*. 42 WT and 42 apolipoprotein E knockout mouse aortas were scanned. The walls, lumen, and plaque objects were segmented using dual-threshold algorithms. Aortic and plaque volumes were computed by voxel counting and lesion surface by triangulation. The results were validated against manual and histological evaluations. Knockout mice had a significant increase in plaque volume compared to wild types with a plaque to aorta volume ratio of 0.3%, 2.8%, and 9.8% at weeks 13, 18, and 26, respectively. Automatic segmentation correlated with manual (*r*^2^ ≥ 0.89; *p* < .001) and histological evaluations (*r*^2^ > 0.96; *p* < .001).

**Conclusions:**

The semiautomatic workflow enabled rapid quantification of atherosclerotic plaques in mice with minimal manual work.

## 1. Introduction

The *ApoE^−/−^* mouse is the most commonly used animal model for studying atherosclerosis. Its primary advantages include ease in handling and rapid formation of the disease [1]. Histological methods are the reference for quantifying atherosclerosis in preclinical models [2]. The aortic root (Paigen) method is predominantly used [3], although only a limited area of the aorta is analyzed; alternately, the *en face* method can evaluate lesions along the whole aorta [4]. Both methods are time-consuming and costly. Adequate characterization of the heterogeneity of atherosclerotic lesions within the aorta would, in fact, require a tight series of tissue sections, but a true three-dimensional method would be the obvious alternative.

MicroCT is a high-resolution, three-dimensional imaging tool commonly available in preclinical labs [5]. Since 1998, microCT was used to assess vasculature [6] and quantify atherosclerosis lesions in autopsy specimens [7], but image quality was limited by poor contrast between tissues. Contrast agent-enhanced microCT can overcome this issue, whereby visualization of atherosclerosis in *ApoE^−/−^* mice with microCT has been achieved with phosphotungstic acid (PTA) [8]. Lloyd et al. validated a quantification method with PTA-enhanced microCT against *en face* histology and found excellent agreement for both plaque surface area and plaque volume [9]. In their study, however, image processing was outsourced and the article did not provide sufficient detail to ensure methodological reproducibility.

Our aim was to develop an automated image processing workflow based on PTA-enhanced microCT scans for the volumetric quantification of atherosclerotic plaques and lesions in entire *ApoE^−/−^* mouse aortas. Further work involved validating this method against a histology reference, manual segmentation, and previous preclinical data as well as describing it in sufficient detail for reproducibility.

## 2. Materials and Methods

### 2.1. Experimental Protocols

For this study, we analyzed aortas from 42 *ApoE^−/−^* and 42 control (WT) C57BL/6 female mice (Jackson Laboratories, Bar Harbor, ME). The protocol for the care and use of our study animals was reviewed and approved by the Charles River Institutional Animal Care and Use Committee and conducted under guidance from the USA National Research Council and Canadian Council on Animal Care.

The animals were housed under standardized conditions and placed on an *ad libitum* high-fat diet (TD.88137, 42% from fat) (Envigo RMS, Indianapolis, IN) starting at 6 weeks old to ensure steady plaque development throughout the study period [10]. All animals were ovariectomized at 8 weeks following standardized protocols [11]. They were euthanized at 13, 18, and 26 weeks of age (*n* = 14 mice/group/time point) under isoflurane anesthesia and whole-body cardiac perfusion as described elsewhere [12].

The aortas *in situ* (remaining attached to the heart, ribs, vertebrae, and kidneys) were then dissected and kept in 10% neutral-buffered formalin for 5 ± 1 days at room temperature during which time all perivascular adipose tissue was removed. Aortas were stained in a 5% PTA solution (Sigma-Aldrich, Oakville, Canada) for 48 ± 2 hours, rinsed 3 times for 60 ± 10 minutes in phosphate-buffered saline, and wrapped in plastic film to avoid evaporation of the reagent during scanning.

### 2.2. MicroCT

#### 2.2.1. Image Acquisition

Each PTA-stained sample was positioned vertically in a ⌀ 19 mm holder and scanned in a cabinet cone-beam microCT scanner (uCT100; Scanco Medical, Brüttisellen, Switzerland). The X-ray tube was operated at 55 kVp/145 *μ*A with a 0.5 mm aluminum filter. Exposure time was 1000 ms/projection for 1000 projections/180°. The slices were reconstructed into 2048 × 2048 voxel images with a 10 *μ*m voxel size (scan volume: 13 × 20.4 × 20.4 mm). Before further processing, images were cropped to a volume of interest from the aortic arch (using valves as landmarks) down to the major portion of the descending arch.

### 2.3. Image Processing Workflow

We developed a semiautomatic workflow to segment aorta wall, lumen, atherosclerotic plaques, and lesions from PTA-enhanced microCT scans. The algorithms were designed primarily to quantify plaques and lesions with a minimum amount of human intervention and be processed natively on the microCT computer system (without further data export or conversions). The workflow comprises five steps: (1) manual contouring of arterial region of interest (ROI), (2) automatic segmentation of walls and lumen, (3) manual verification of the lumen contour (and correction as necessary), (4) automatic segmentation of plaques, and (5) automatic quantification of plaques and lesions and three-dimensional rendering. The algorithms were developed using EasyIPL (http://easyipl.com), a high-level library of macros using the scanner software IPL (Scanco Medical AG, Brüttisellen, Switzerland) and the scripting language DCL of the scanner operating system, OpenVMS (Hewlett-Packard, Palo Alto, CA).

#### 2.3.1. Manual and Autocontouring Process

**Figure 1:**
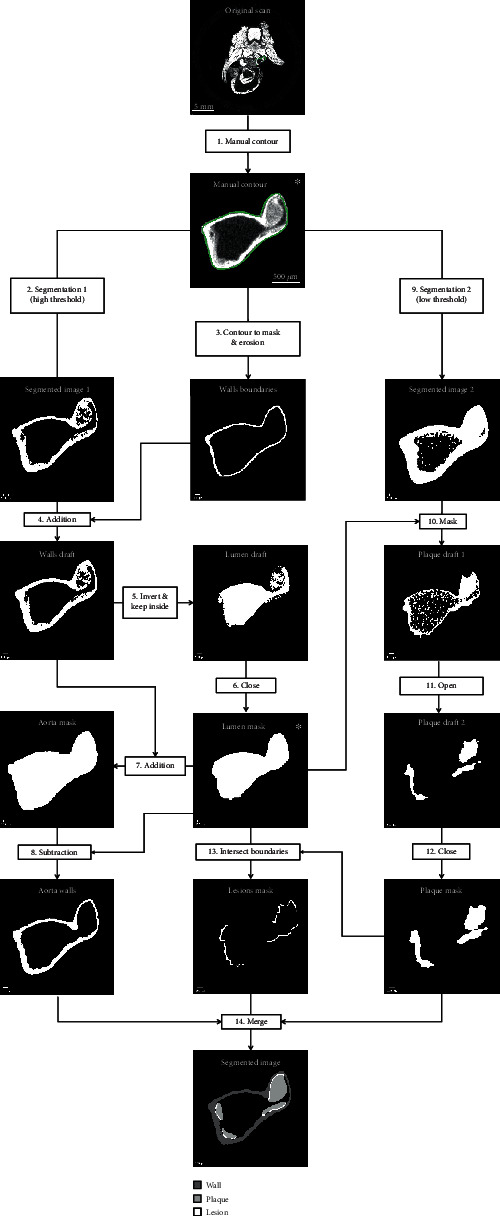
Representation of the segmentation algorithm. (a) Cropping to ROI. (b) High threshold to create aortic wall draft. (c) Manual contour converted into a mask. (d) Inward dilation by two voxels and juxtaposition to wall draft. (e) Inversion and inner volume extracted as lumen draft. (f) Dilation/erosion (5 voxels) to remove plaque from lumen draft. (g) Addition of lumen mask and wall draft. (h) Subtraction of lumen generates wall mask. (i) Plaque segmentation: Gaussian filter (*σ* = 0.8; support = 1) then low threshold (*μ* = 2.0 cm^−1^). (j) Lumen mask applied. (k) Refinement with opening (*d* = 1 voxel; minimum volume = 2% total volume) followed by (l) closing (*d* = 3 voxels) filters. (m) Aortic lesions defined as contacts between plaque and wall. (n) Aortic wall, plaque, and lesion concatenated. ∗ indicates manual interventions.

The first two workflow steps define a mask of the aortic lumen, which is later used as a ROI for plaque segmentation. Firstly, the aortic ROI—comprising the ascending aorta starting from the valves, the aortic arch, and 1 cm of the descending aorta caudal to the ostium of the left subclavian artery—is manually identified. The outer contour of the aorta is manually drawn at regular intervals (typically 10 contours over the specimen length), and the full ROI is then interpolated automatically (Figure 1(a)).

In a second step, a fixed threshold (*μ* = 6.0 cm^−1^) is applied to the grayscale data within the aortic ROI to generate a draft of the aortic walls (Figure 1(b)). A closed aortic wall mask is necessary to define the lumen at a later stage, but the draft can be imperfect if highly stained plaques are attached to the wall or if insufficiently stained wall regions and arterial branches create discontinuities in the walls. The aortic wall draft is therefore closed by eroding the aortic ROI by two voxels and overlaying it to the draft walls (Figures 1(c) and 1(d)). This new object is inverted, and the inside volume is used as a draft of the lumen (Figure 1(e)). Sometimes highly stained plaques can be confounded with the wall during thresholding, which leaves voids in the lumen draft; these are eliminated using a 5-voxel dilation/erosion sequence (Figure 1(f)). At this point, the operator can verify the lumen mask and make small adjustments as required. Finally, the aorta mask is generated by adding the aortic wall draft and lumen mask (Figure 1(g)), and the final wall mask is obtained by subtracting the lumen mask from the aorta mask (Figure 1(h)).

#### 2.3.2. Plaque and Lesion Segmentation

A Gaussian filter (*σ* = 0.8; support = 1) and a threshold of 2.0 cm^−1^ are applied within the lumen mask (Figure 1(i)) to segment all stained tissues inside the aortic lumen (Figure 1(j)). This plaque draft is refined using an opening (distance = 1 voxel; minimum volume = 2% total volume) and then a closing filter (distance = 3 voxels) (Figures 1(k) and 1(l)). Aortic lesions are defined as the regions of contact between plaques and aortic wall (Figure 1(m)). Aortic wall, plaques, and lesions are finally concatenated to form a model for three-dimensional rendering and volumetric quantification (Figure 1(n)).

#### 2.3.3. Volumetric Quantification

Lumen and plaque volumes are quantified by direct voxel counting of the respective masks. The arterial wall inner surface is computed by triangulation of the lumen mask [13] and arterial wall thickness by a distance transform algorithm applied to the wall mask [14]. The lesion surface is calculated as 50% of the triangulated lesion mask surface (i.e., only one side of a quasi-planar object).

(1) $$\begin{array}{c} \text{Plaque volume fraction }\left(\%\right)=100*\frac{\text{Plaque volume }\left({\text{mm}}^{3}\right)}{\text{Lumen volume}\left({\text{mm}}^{3}\right)},\\ \text{Lesion surface fraction }\left(\%\right)=100*\frac{\text{Lesion surface }\left({\text{mm}}^{2}\right)}{\text{Lumen surface }\left({\text{mm}}^{2}\right)}. \end{array}$$

### 2.4. Histology

After microCT scanning, PTA-treated tissues were soaked for one week in 4% paraformaldehyde/7.5% sucrose fixative to remove PTA. Aortas were separated from the heart and other tissues, embedded in paraffin, and separated into single blocks of the ascending aorta, aortic arch, and descending thoracic aorta for sectioning and hematoxylin-eosin staining as recommended by the American Heart Association [15]. Histopathological evaluation was performed by a board-certified veterinary pathologist using the classification of Stary et al. [16].

### 2.5. Validations of the Semi-automatic Method

#### 2.5.1. Validation against Manual Segmentation in MicroCT

Automatic segmentations were compared to manual contouring in 14 microCT sections with plaque (from *ApoE^−/−^* mice at 18 and 26 weeks). The corresponding grayscale and segmented slices were exported to Fiji (ImageJ) [17] and evaluated independently. The lumen, plaques, and lesions were manually identified in each slice, and the lengths and areas of these contours were measured using the integrated tools. The procedure was repeated three times for each slice, and average values were used to calculate stenosis and lesion length expressed as a percentage of the lumen area and perimeter, respectively:

(2) $$\begin{array}{c} \text{Stenosis }\left(\%\right)=100*\frac{\text{Plaque area }\left({\text{mm}}^{2}\right)}{\text{Lumen area }\left({\text{mm}}^{2}\right)},\\ \text{Lesion length }\left(\%\right)=100*\frac{\text{Lesion lengh }\left(\text{mm}\right)}{\text{Lumen perimeter }\left(\text{mm}\right)}. \end{array}$$

#### 2.5.2. Validation against Histological Quantification

Automatic segmentations were compared to histological quantifications in 15 slices. To achieve spatially matching images, the gross position and orientation of the histological section in a microCT volume were manually identified. Anatomical features from histological sections were searched visually in corresponding microCT scans. The landmark coordinates were used to rotate the scans rigidly and register them within the histology coordinate system. The process was repeated until the registration was deemed qualitatively satisfactory (*r*^2^ > 0.9). The histological and corresponding microCT segmented sections were exported to Fiji for evaluation of the lumen, plaques, and lesions as described above.

### 2.6. Statistical Analysis

Data analyses were implemented using R (version 3.5.2) [18]. Data were expressed as the mean and standard deviation unless stated otherwise. Correlations between manual and automatic outcomes were evaluated by fitting linear models to the data and using Bland-Altman plots to detect bias [19]. *ApoE^−/−^* and WT groups were compared with one-way ANOVA, and *p* < .05 was considered statistically significant.

## 3. Results

### 3.1. Validations

#### 3.1.1. Validation against Manual Segmentation in MicroCT

**Figure 2:**
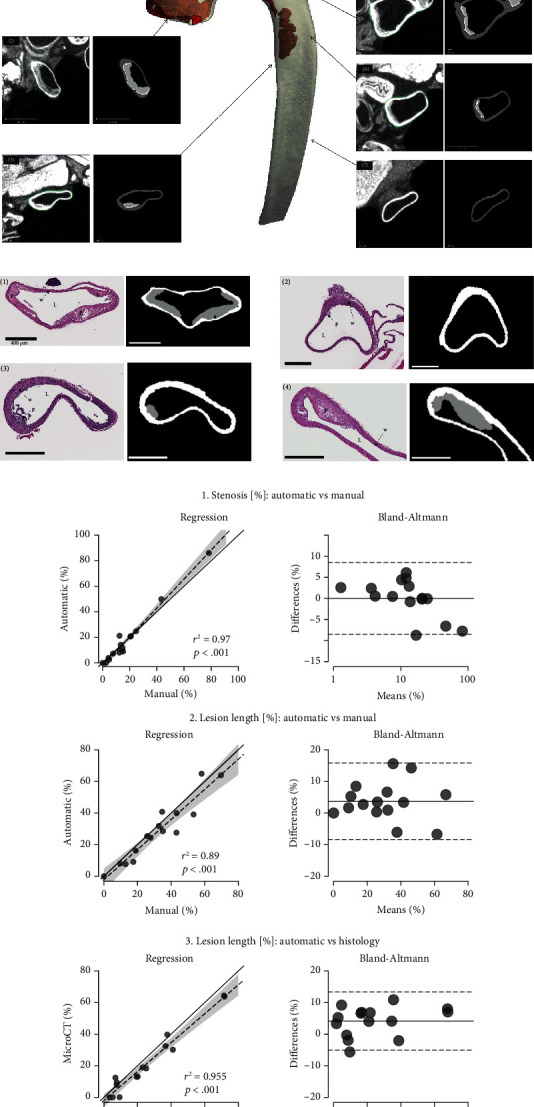
Validation against manual and histopathological measurements. (a) Seven representative sections for visual comparison of grayscale and automatically segmented microCT sections along the aorta illustrating excellent qualitative agreement. (b) Four representative pairs of equivalent microCT and histological slices (hematoxylin-eosin staining) aligned using rigid registration illustrating the good agreement between methods (p: plaque; L: lumen; w: wall; bar = 400 *μ*m). (c) Quantitative analysis: regression and Bland-Altman plots showing very good agreement between (1 and 2) automatic and manual measurements and no bias (*r*^2^ ≥ 0.89; *p* < .001) and (3) very good agreement between microCT and histology (*r*^2^ > 0.95; *p* < 0.001) with slightly lower (~5%) measurements in microCT, but evenly distributed errors suggest no bias.

Visual comparison of the automatic segmented plaques revealed very good correspondence with the manually contoured plaques (Figure 2(a)). In addition, automatic and manual measurements of stenosis and lesion length correlated strongly (*r*^2^ ≥ 0.89; *p* < .001) with only minor underestimation (3%) of lesion lengths in the automatic method; Bland-Altman plots showed evenly distributed errors, and all points were within two standard deviations suggesting good agreement between methods (Figure 2(c)).

#### 3.1.2. Validation against Histological Quantification

A visual comparison of the automatic segmented plaques revealed very good correspondence with the histologic sections (Figure 2(b)). Automatic microCT measurements correlated strongly with histology lesion length (*r*^2^ > 0.96), although there was a slight (~5%) underestimation (Figure 2(c)). Bland-Altman plots also showed this underestimation, but errors were evenly distributed suggesting good agreement between methods.

### 3.2. Development of Atherosclerotic Lesions in *ApoE^−/−^* and WT Mice

**Figure 3:**
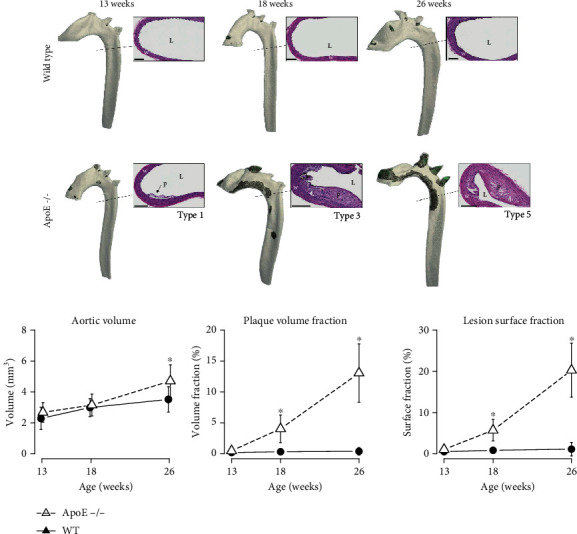
Development of aortic atherosclerosis in *ApoE^−/−^* and WT mice. (a) Three-dimensional renderings and corresponding histological (hematoxylin-eosin) sections showing plaque detection at 13, 18, and 26 weeks of age. Bars = 100 *μ*m, and the cut plane is shown with dotted lines (p: plaque; L: lumen; w: wall). Animals with median plaque volume are shown. WT mice presented no atherosclerotic lesions, while *ApoE^−/−^* mice showed progressively larger plaques in the aortic root, lesser curvature of the aortic arch, and principal branches of the aorta over time. (b) Quantitative evolution of aortic volume, plaque volume fraction, and lesion surface fraction in *ApoE^−/−^* (triangles/dashed lines) and WT (dots/plain lines) mice. Aortic volumes are comparable between the two groups, but *ApoE^−/−^* mice show significantly larger plaques and lesions. ^∗^*p* < .05.

At 13 weeks of age, there were no clinically significant plaques in both WT and *ApoE^−/−^* mice (Figure 3(a)). With age, *ApoE^−/−^* mice developed atherosclerotic plaques, principally in the aortic root, the lesser curvature of the aortic arch, and principal branches of the aorta. Based on histological characterization, *ApoE^−/−^* mice had mainly Stary type 1 plaques at 13 weeks and type 3 (with intraplaque fibro-cartilaginous tissue) and type 5 lesions at 18 and 26 weeks, respectively.

Aortic volume increased with age in *ApoE^−/−^* and WT mice with similar volumes measured at 13 and 18 weeks of age (2.5 ± 0.7 mm^3^ and 3.0 ± 0.6 mm^3^), but at 26 weeks, *ApoE^−/−^* mice had significantly larger aortic volumes (4.7 ± 1.0 mm^3^) than WT mice (3.5 ± 0.8 mm^3^; *p* = .005). There was a continuous increase in plaque volume (rate = 0.05 ± 0.01 mm^3^/week) and lesion surface (rate = 0.63 ± 0.09 mm^2^/week) for *ApoE^−/−^* mice between 13 and 26 weeks, which was significantly different from the lack of any notable growth in plaque volume (rate = 0.001 ± 0.0003 mm^3^/week) or lesion surface (rate = 0.012 ± 0.009 mm^2^/week) in WT mice (*p* < .001). *ApoE^−/−^* mice had significantly more plaque volume and lesion surface at 18 and 26 weeks of age compared to WT animals (*p* < .001) (Figure 3(b)).

## 4. Discussion

Contrast-enhanced microCT imaging in atherosclerotic mouse models is gaining momentum [20] and is even used as a reference for validating new modalities including micro-MRI or micro-SPECT [20, 21]. However, plaque quantification is still mostly performed manually [21, 22]. Only one semiautomatic segmentation method has been formally validated, but the technical details were not revealed [9]. We developed a 5-step workflow for quantifying atherosclerotic plaques through the entire aorta via a series of algorithms for automatic segmentation. These algorithms run natively from the scanning interface to enable efficient and reproducible evaluations. Automatic segmentation correlated highly with manual and histological evaluations on matching sections. *ApoE^−/−^* mice had continuous atherosclerotic plaque growth in volume and lesion surface from age 13 to 26 weeks; these measurements correlated well with the histopathology analysis grading. In contrast, WT mice did not develop any plaques.

The outcomes of our semiautomatic workflow concur with that expected of the *ApoE^−/−^* model, whereby detectable plaque formation occurred from age 18 weeks onwards and the progression rate is consistent with previous findings [22, 23]. We detected plaques in the predilection sites, i.e. “the aortic root, the lesser curvature of the aortic arch, and the principal branches of the aorta,” as described by Nakashima et al. [24].

In terms of the technical aspects of our procedure, our algorithms could not be directly compared to those of Lloyd et al. [9] due to their lack of methodological disclosure and reliance on an external company for the analysis. In contrast, our workflow was designed to run natively on the scanner computer system, which spares the operator from converting and transferring large amounts of data (~5 GB for a single scan and 400 GB for 84 scans). We did not formally evaluate the gain in time compared to manual analysis of up to a thousand sections for a single aorta. Nevertheless, we can speculate that the gain is above 10-fold with the automated workflow based on previous experience.

Although most processes were automatic, some technically challenging scenarios still required manual input. For example, discontinuities in the aortic wall due to arterial branches needed to be closed during initial contouring and/or postprocessing of collapsed specimens during tissue preparation was required when the lumen was very narrow, and partial volume effects seen in the wall were confounded with plaque. These interventions naturally increase the time needed for the entire process, yet eliminating these interventions via additional algorithms poses a major technical challenge given the myriad of scenarios associated with sampling and processing tissue specimens.

Manual versus automatic segmentations were performed on a limited number of single matching sections. Still, the very high correlations in single sections suggest the volume comparisons would also have been very high. We also compared only a limited number of scans with histology sections for efficiency and cost purposes. As expected with landmark-based rigid registration strategy, we did not always establish a perfectly corresponding alignment between microCT and histology sections. The registration of three- and two-dimensional images is a largely unsolved problem and is complicated by the deformation/damage encountered during tissue processing as well as the varied appearances of landmarks between modalities [25]. In this context, the correlations measured from 15 matching pairs were high enough to support the equivalence between microCT and histology.

The presented workflow was validated using the OVX *ApoE^−/−^* mouse model, in the context of a nonclinical cardiovascular safety evaluation of the sclerostin inhibitor *romosozumab* [26]. OVX accelerates aortic atherosclerotic lesion development due to lack of cardio-protective estrogen but not lesion distribution [27]; therefore, we assume this method is applicable to other models such as ovary-intact *ApoE^−/−^* or LDLR−/−, the low-density lipoprotein receptor knockout mouse [28]. We also speculate that this workflow may be applicable to other imaging modalities with similar resolution and contrast, after adjusting the segmentation parameters. Finally, the workflow was only implemented on a Scanco microCT system, but we cannot foresee any major challenges porting the scripts to other platforms.

We were able to automatically quantify atherosclerotic plaque volume and lesion surfaces in *ApoE^−/−^* mice. The results, validated against histology and manual measurements, were consistent with the expected development of atherosclerosis in this model. The semiautomatic workflow for quantification of atherosclerotic plaques and lesions in contrast-enhanced microCT scans of mouse aortas enables researchers to perform their analysis independently. In this workflow, human input is minimized to enable better reproducibility and higher throughput. MicroCT can be used for spatial distribution and volumetric quantification of plaques and lesions in a nondestructive fashion, enabling to perform histopathology to selected sections for plaque grading, which saves a significant amount of time and effort.

## Data Availability

The data used to support the findings of this study are available from the corresponding author upon reasonable request.

## Abbreviations

*ApoE^−^/^−^*: Apolipoprotein E knockout
PTA: Phosphotungstic acid
ROI: Region of interest
WT: Wild type/control.

## Additional Points

*Highlights.* (1) A semiautomatic image processing workflow was developed based on phosphotungstic acid- (PTA-) enhanced microCT scans for the volumetric quantification of atherosclerosis in mouse aortas. (2) Automatic measurements of plaque area and lesion length correlated strongly with manual measurements made in grayscale microCT (*r*^2^ ≥ 0.89; *p* < .001) and hematoxylin-eosin-stained slices (*r*^2^ > 0.95; *p* < .001). (3) The automatic method enables monitoring and localization of atherosclerosis in mice more efficiently and without the inconvenience associated with outsourcing.
